# Crystal Structures of Putative Sugar Kinases from *Synechococcus Elongatus* PCC 7942 and *Arabidopsis Thaliana*

**DOI:** 10.1371/journal.pone.0156067

**Published:** 2016-05-25

**Authors:** Yuan Xie, Mei Li, Wenrui Chang

**Affiliations:** 1 National Laboratory of Biomacromolecules, CAS Center for Excellence in Biomacromolecules, Institute of Biophysics, Chinese Academy of Sciences, Beijing, China; 2 University of Chinese Academy of Sciences, Beijing, China; University of Roskilde, DENMARK

## Abstract

The genome of the *Synechococcus elongatus* strain PCC 7942 encodes a putative sugar kinase (SePSK), which shares 44.9% sequence identity with the xylulose kinase-1 (AtXK-1) from *Arabidopsis thaliana*. Sequence alignment suggests that both kinases belong to the ribulokinase-like carbohydrate kinases, a sub-family of FGGY family carbohydrate kinases. However, their exact physiological function and real substrates remain unknown. Here we solved the structures of SePSK and AtXK-1 in both their apo forms and in complex with nucleotide substrates. The two kinases exhibit nearly identical overall architecture, with both kinases possessing ATP hydrolysis activity in the absence of substrates. In addition, our enzymatic assays suggested that SePSK has the capability to phosphorylate D-ribulose. In order to understand the catalytic mechanism of SePSK, we solved the structure of SePSK in complex with D-ribulose and found two potential substrate binding pockets in SePSK. Using mutation and activity analysis, we further verified the key residues important for its catalytic activity. Moreover, our structural comparison with other family members suggests that there are major conformational changes in SePSK upon substrate binding, facilitating the catalytic process. Together, these results provide important information for a more detailed understanding of the cofactor and substrate binding mode as well as the catalytic mechanism of SePSK, and possible similarities with its plant homologue AtXK-1.

## Introduction

Carbohydrates are essential cellular compounds involved in the metabolic processes present in all organisms. Phosphorylation is one of the various pivotal modifications of carbohydrates, and is catalyzed by specific sugar kinases. These kinases exhibit considerable differences in their folding pattern and substrate specificity. Based on sequence analysis, they can be divided into four families, namely HSP 70_NBD family, FGGY family, Mer_B like family and Parm_like family. The FGGY family carbohydrate kinases contain different types of sugar kinases, all of which possess different catalytic substrates with preferences for short-chained sugar substrates, ranging from triose to heptose. These sugar substrates include L-ribulose, erythritol, L-fuculose, D-glycerol, D-gluconate, L-xylulose, D-ribulose, L-rhamnulose and D-xylulose [1]. Structures reported in the Protein Data Bank of the FGGY family carbohydrate kinases exhibit a similar overall architecture containing two protein domains, one of which is responsible for the binding of substrate, while the second is used for binding cofactor ATP. While the binding pockets for substrates are at the same position, each FGGY family carbohydrate kinases uses different substrate-binding residues, resulting in high substrate specificity [2–4].

Synpcc7942_2462 from the cyanobacteria *Synechococcus elongatus* PCC 7942 encodes a putative sugar kinase (SePSK), and this kinase contains 426 amino acids. The At2g21370 gene product from *Arabidopsis thaliana*, xylulose kinase-1 (AtXK-1), whose mature form contains 436 amino acids, is located in the chloroplast (ChloroP 1.1 Server) [5, 6]. SePSK and AtXK-1 display a sequence identity of 44.9%, and belong to the ribulokinase-like carbohydrate kinases, a sub-family of FGGY family carbohydrate kinases. Members of this sub-family are responsible for the phosphorylation of sugars similar to L-ribulose and D-ribulose. The sequence and the substrate specificity of ribulokinase-like carbohydrate kinases are different, but they share the common folding feature with two domains. Domain I exhibits a ribonuclease H-like folding pattern, and is responsible for the substrate binding, while domain II possesses an actin-like ATPase domain that binds cofactor ATP [1–4, 7–9].

Two possible xylulose kinases (xylulose kinase-1: XK-1 and xylulose kinase-2: XK-2) from *Arabidopsis thaliana* were previously proposed [6]. It was shown that XK-2 (At5g49650) located in the cytosol is indeed xylulose kinase. However, the function of XK-1 (At2g21370) inside the chloroplast stroma has remained unknown. SePSK from *Synechococcus elongatus* strain PCC 7942 is the homolog of AtXK-1, though its physiological function and substrates remain unclear. In order to obtain functional and structural information about these two proteins, here we reported the crystal structures of SePSK and AtXK-1. Our findings provide new details of the catalytic mechanism of SePSK and lay the foundation for future studies into its homologs in eukaryotes.

## Results and Discussion

### Overall structures of apo-SePSK and apo-AtXK-1

The attempt to solve the SePSK structure by molecular replacement method failed with ribulokinase from *Bacillus halodurans* (PDB code: 3QDK, 15.7% sequence identity) as an initial model [3]. We therefore used single isomorphous replacement anomalous scattering method (SIRAS) for successful solution of the apo-SePSK structure at a resolution of 2.3 Å. Subsequently, the apo-SePSK structure was used as molecular replacement model to solve all other structures identified in this study.

**Fig 1:**
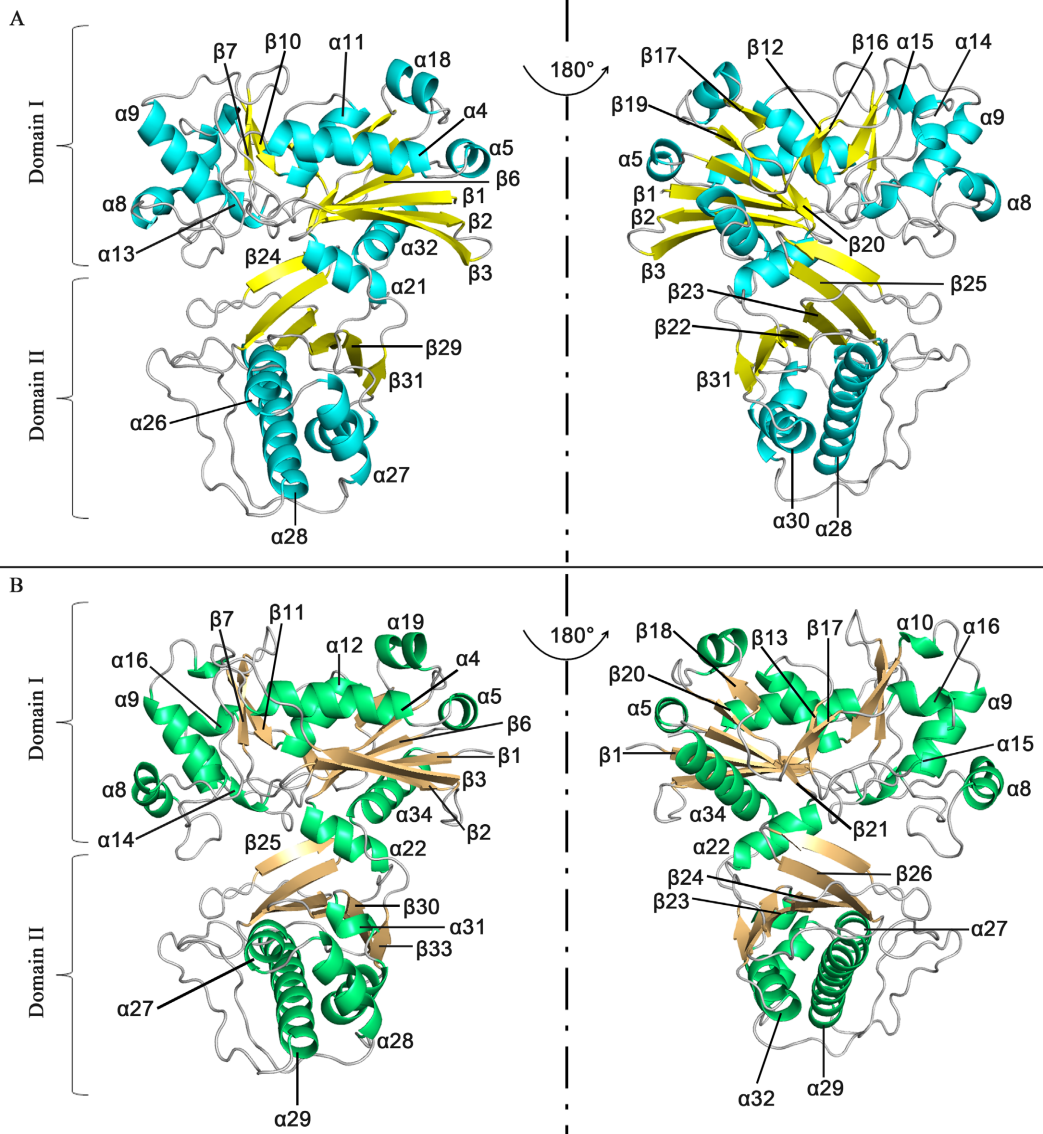
Overall structures of SePSK and AtXK-1. (A) Three-dimensional structure of apo-SePSK. The secondary structural elements are indicated (α-helix: cyan, β-sheet: yellow). (B) Three-dimensional structure of apo-AtXK-1. The secondary structural elements are indicated (α-helix: green, β-sheet: wheat).

Our structural analysis showed that apo-SePSK consists of one SePSK protein molecule in an asymmetric unit. The amino-acid residues were traced from Val2 to His419, except for the Met1 residue and the seven residues at the C-termini. Apo-SePSK contains two domains referred to further on as domain I and domain II (Fig 1A). Domain I consists of non-contiguous portions of the polypeptide chains (aa. 2–228 and aa. 402–419), exhibiting 11 α-helices and 11 β-sheets. Among all these structural elements, α4/α5/α11/α18, β3/β2/β1/β6/β19/β20/β17 and α21/α32 form three patches, referred to as A1, B1 and A2, exhibiting the core region. In addition, four β-sheets (β7, β10, β12 and β16) and five α-helices (α8, α9, α13, α14 and α15) flank the left side of the core region. Domain II is comprised of aa. 229–401 and classified into B2 (β31/β29/β22/β23/β25/β24) and A3 (α26/α27/α28/α30) (Fig 1A and S1 Fig). In the SePSK structure, B1 and B2 are sandwiched by A1, A2 and A3, and the whole structure shows the A1/B1/A2/B2/A3 (α/β/α/β/α) folding pattern, which is in common with other members of FGGY family carbohydrate kinases (S2 Fig) [3, 4, 10]. The overall folding of SePSK resembles a clip, with A2 of domain I acting as a hinge region. As a consequence, a deep cleft is formed between the two domains.

Apo-AtXK-1 exhibits a folding pattern similar to that of SePSK in line with their high sequence identity (Fig 1B and S1 Fig). However, superposition of structures of AtXK-1 and SePSK shows some differences, especially at the loop regions. A considerable difference is found in the loop3 linking β3 and α4, which is stretched out in the AtXK-1 structure, while in the SePSK structure, it is bent back towards the inner part. The corresponding residues between these two structures (SePSK-Lys35 and AtXK-1-Lys48) have a distance of 15.4 Å (S3 Fig).

### Activity assays of SePSK and AtXK-1

**Fig 2:**
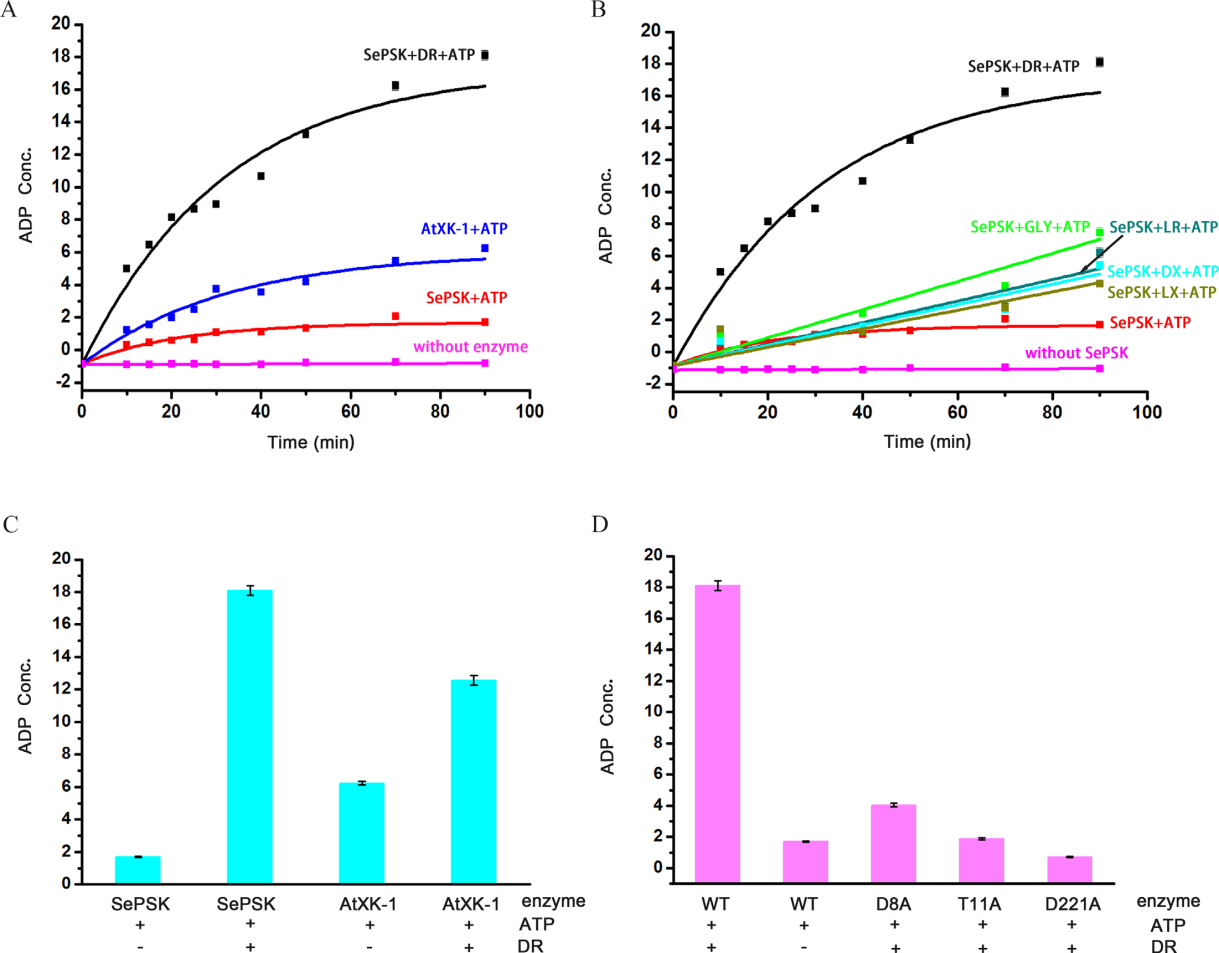
The enzymatic activity assays of SePSK and AtXK-1. (A) The ATP hydrolysis activity of SePSK and AtXK-1. Both SePSK and AtXK-1 showed ATP hydrolysis activity in the absence of substrate. While the ATP hydrolysis activity of SePSK greatly increases upon addition of D-ribulose (DR). (B) The ATP hydrolysis activity of SePSK with addition of five different substrates. The substrates are DR (D-ribulose), LR (L-ribulose), DX (D-xylulose), LX (L-xylulose) and GLY (Glycerol). (C) The ATP hydrolysis activity of SePSK and AtXK-1 with or without D-ribulose. (D) The ATP hydrolysis activity of wild-type (WT) and single-site mutants of SePSK. Three single-site mutants of SePSK are D8A-SePSK, T11A-SePSK and D221A-SePSK. The ATP hydrolysis activity measured via luminescent ADP-Glo assay (Promega).

In order to understand the function of these two kinases, we performed structural comparison using Dali server. The structures most closely related to SePSK are xylulose kinase, glycerol kinase and ribulose kinase, implying that SePSK and AtXK-1 might function similarly to these kinases. We first tested whether both enzymes possessed ATP hydrolysis activity in the absence of substrates. As shown in Fig 2A, both SePSK and AtXK-1 exhibited ATP hydrolysis activity. This finding is in agreement with a previous result showing that xylulose kinase (PDB code: 2ITM) possessed ATP hydrolysis activity without adding substrate [4]. To further identify the actual substrate of SePSK and AtXK-1, five different sugar molecules, including D-ribulose, L-ribulose, D-xylulose, L-xylulose and Glycerol, were used in enzymatic activity assays. As shown in Fig 2B, the ATP hydrolysis activity of SePSK greatly increased upon adding D-ribulose than adding other potential substrates, suggesting that it has D-ribulose kinase activity. In contrary, limited increasing of ATP hydrolysis activity was detected for AtXK-1 upon addition of D-ribulose (Fig 2C), despite its structural similarity with SePSK.

To understand the catalytic mechanism of SePSK, we performed structural comparisons among xylulose kinase, glycerol kinase, ribulose kinase and SePSK. Our results suggested that three conserved residues (D8, T11 and D221 of SePSK) play an important role in SePSK function. Mutations of the corresponding residue in xylulose kinase and glycerol kinase from *Escherichia coli* greatly reduced their activity [4, 11]. To identify the function of these three residues of SePSK, we constructed D8A, T11A and D221A mutants. Using enzymatic activity assays, we found that all of these mutants exhibit much lower activity of ATP hydrolysis after adding D-ribulose than that of wild type, indicating the possibility that these three residues are involved in the catalytic process of phosphorylation D-ribulose and are vital for the function of SePSK (Fig 2D).

### SePSK and AtXK-1 possess a similar ATP binding site

To obtain more detailed information of SePSK and AtXK-1 in complex with ATP, we soaked the apo-crystals in the reservoir adding cofactor ATP, and obtained the structures of SePSK and AtXK-1 bound with ATP at the resolution of 2.3 Å and 1.8 Å, respectively. In both structures, a strong electron density was found in the conserved ATP binding pocket, but can only be fitted with an ADP molecule (S4 Fig). Thus the two structures were named ADP-SePSK and ADP-AtXK-1, respectively. The extremely weak electron densities of ATP γ-phosphate in both structures suggest that the γ-phosphate group of ATP is either flexible or hydrolyzed by SePSK and AtXK-1. This result was consistent with our enzymatic activity assays where SePSK and AtXK-1 showed ATP hydrolysis activity without adding any substrates (Fig 2A and 2C).

**Fig 3:**
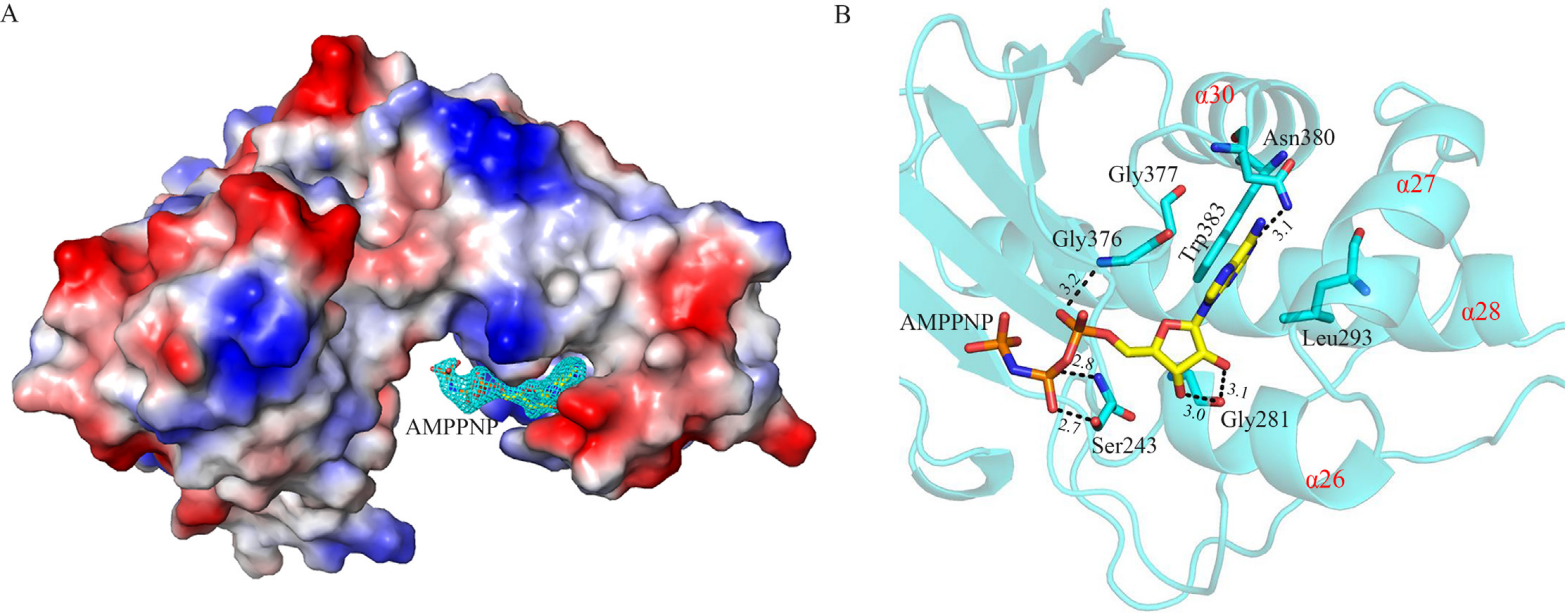
Structure of SePSK in complex with AMP-PNP. (A) The electron density of AMP-PNP. The SePSK structure is shown in the electrostatic potential surface mode. The AMP-PNP is depicted as sticks with its *ǀFoǀ-ǀFcǀ* map contoured at 3 σ shown as cyan mesh. (B) The AMP-PNP binding pocket. The head of AMP-PNP is sandwiched by four residues (Leu293, Gly376, Gly377 and Trp383). The protein skeleton is shown as cartoon (cyan). The four α-helices (α26, α28, α27 and α30) are labeled in red. The AMP-PNP and coordinated residues are shown as sticks. The interactions between them are represented as black dashed lines. The numerical note near the black dashed line indicates the distance (Å).

To avoid hydrolysis of ATP, we soaked the crystals of apo-SePSK and apo-AtXK-1 into the reservoir adding AMP-PNP. However, we found that the electron densities of γ-phosphate group of AMP-PNP (AMP-PNP γ-phosphate) are still weak in the AMP-PNP-SePSK and AMP-PNP-AtXK-1 structures, suggesting high flexibility of ATP-γ-phosphate. The γ-phosphate group of ATP is transferred to the sugar substrate during the reaction process, so this flexibility might be important for the ability of these kinases. The overall structures as well as the coordination modes of ADP and AMP-PNP in the AMP-PNP-AtXK-1, ADP-AtXK-1, ADP-SePSK and AMP-PNP-SePSK structures are nearly identical (S5 Fig), therefore the structure of AMP-PNP-SePSK is used here to describe the structural details and to compare with those of other family members. As shown in Fig 3A, one SePSK protein molecule is in an asymmetric unit with one AMP-PNP molecule. The AMP-PNP is bound at the domain II, where it fits well inside a positively charged groove. The AMP-PNP binding pocket consists of four α-helices (α26, α28, α27 and α30) and forms a shape resembling a half-fist (Fig 3A and 3B). The head group of the AMP-PNP is embedded in a pocket surrounded by Trp383, Asn380, Gly376 and Gly377. The purine ring of AMP-PNP is positioned in parallel to the indole ring of Trp383. In addition, it is hydrogen-bonded with the side chain amide of Asn380 (Fig 3B). The tail of AMP-PNP points to the hinge region of SePSK, and its α-phosphate and β-phosphate groups are stabilized by Gly376 and Ser243, respectively. Together, this structure clearly shows that the AMP-PNP-β-phosphate is sticking out of the ATP binding pocket, thus the γ-phosphate group is at the empty space between domain I and domain II and is unconstrained in its movement by the protein.

### The potential substrate binding site in SePSK

The results from our activity assays suggested that SePSK has D-ribulose kinase activity. To better understand the interaction pattern between SePSK and D-ribulose, the apo-SePSK crystals were soaked into the reservoir with 10 mM D-ribulose (RBL) and the RBL-SePSK structure was solved. As shown in S6 Fig, two residual electron densities are visible in domain I, which can be interpreted as two D-ribulose molecules with reasonable fit.

**Fig 4:**
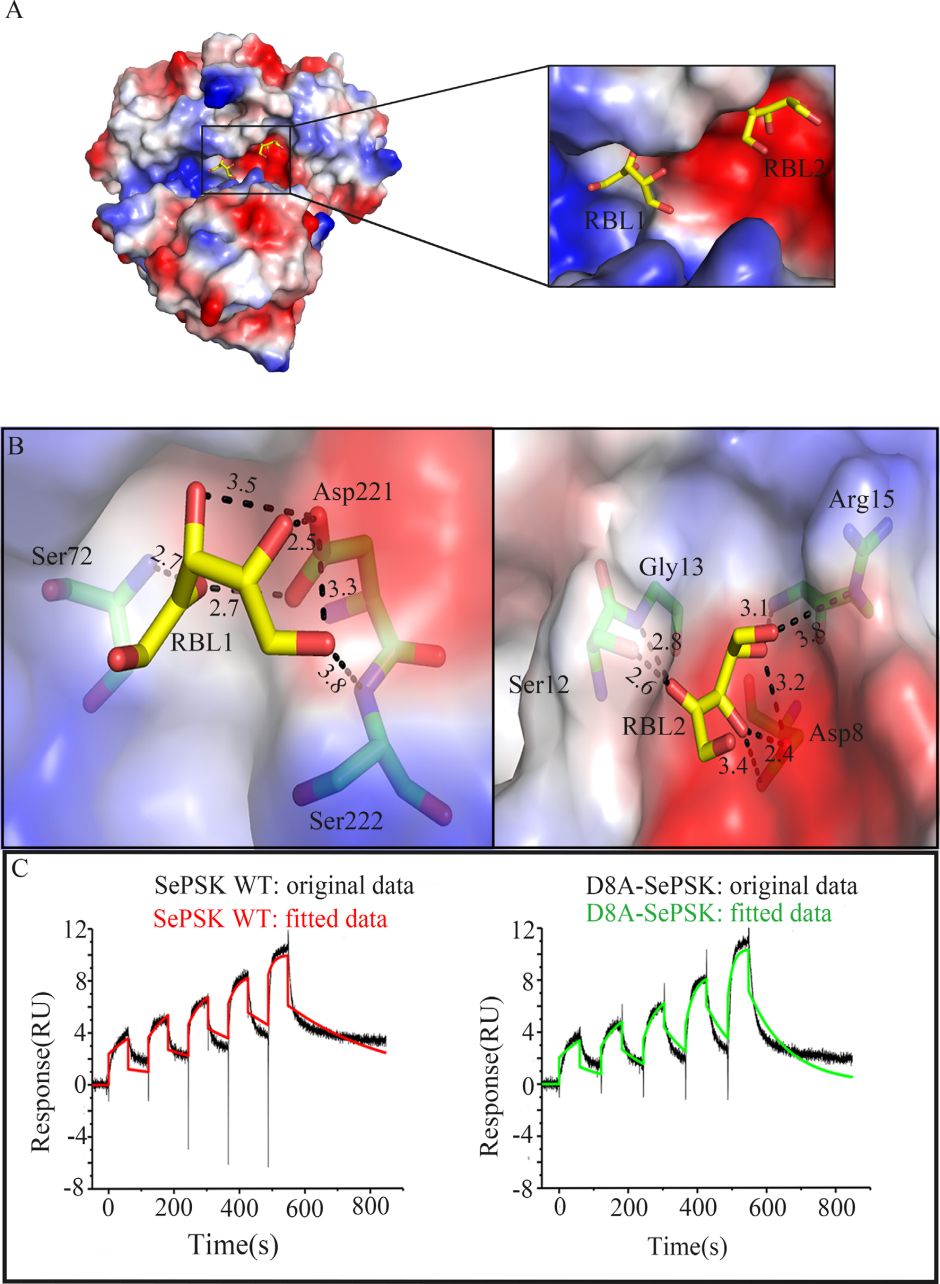
The binding of D-ribulose (RBL) with SePSK. (A) The electrostatic potential surface map of RBL-SePSK and a zoom-in view of RBL binding site. The RBL1 and RBL2 are depicted as sticks. (B) Interaction of two D-ribulose molecules (RBL1 and RBL2) with SePSK. The RBL molecules (carbon atoms colored yellow) and amino acid residues of SePSK (carbon atoms colored green) involved in RBL interaction are shown as sticks. The hydrogen bonds are indicated by the black dashed lines and the numbers near the dashed lines are the distances (Å). (C) The binding affinity assays of SePSK with D-ribulose. Single-cycle kinetic data are reflecting the interaction of SePSK and D8A-SePSK with D-ribulose. It shows two experimental sensorgrams after minus the empty sensorgrams. The original data is shown as black curve, and the fitted data is shown as different color (wild type SePSK: red curve, D8A-SePSK: green curve). Dissociation rate constant of wild type and D8A-SePSK are 3 ms^-1^ and 9 ms^-1^, respectively.

As shown in Fig 4A, the nearest distance between the carbon skeleton of two D-ribulose molecules are approx. 7.1 Å (RBL1-C4 and RBL2-C1). RBL1 is located in the pocket consisting of α21 and the loop between β6 and β7. The O4 and O5 of RBL1 are coordinated with the side chain carboxyl group of Asp221. Furthermore, the O2 of RBL1 interacts with the main chain amide nitrogen of Ser72 (Fig 4B). This pocket is at a similar position of substrate binding site of other sugar kinase, such as L-ribulokinase (PDB code: 3QDK) (S7 Fig). However, structural comparison shows that the substrate ligating residues between the two structures are not strictly conserved. Based on the structures, the ligating residues of RBL1 in RBL-SePSK structure are Ser72, Asp221 and Ser222, and the interacting residues of L-ribulose with L-ribulokinase are Ala96, Lys208, Asp274 and Glu329 (S7 Fig). Glu329 in 3QDK has no counterpart in RBL-SePSK structure. In addition, although Lys208 of L-ribulokinase has the corresponding residue (Lys163) in RBL-SePSK structure, the hydrogen bond of Lys163 is broken because of the conformational change of two α-helices (α9 and α13) of SePSK. These differences might account for their different substrate specificity.

The binding pocket of RBL2 with relatively weak electron density is near the N-terminal region of SePSK and is negatively charged. The side chain of Asp8 interacts strongly with O3 and O4 of RBL2. The hydroxyl group of Ser12 coordinates with O2 of RBL2. The backbone amide nitrogens of Gly13 and Arg15 also keep hydrogen bonds with RBL2 (Fig 4B). Structural comparison of SePSK and AtXK-1 showed that while the RBL1 binding pocket is conserved, the RBL2 pocket is disrupted in AtXK-1 structure, despite the fact that the residues interacting with RBL2 are highly conserved between the two proteins. In the RBL-SePSK structure, a 2.6 Å hydrogen bond is present between RBL2 and Ser12 (Fig 4B), while in the AtXK-1 structure this hydrogen bond with the corresponding residue (Ser22) is broken. This break is probably induced by the conformational change of the two β-sheets (β1 and β2), with the result that the linking loop (loop 1) is located further away from the RBL2 binding site. This change might be the reason that AtXK-1 only shows limited increasing in its ATP hydrolysis ability upon adding D-ribulose as a substrate after comparing with SePSK (Fig 2C).

Our SePSK structure shows that the Asp8 residue forms strong hydrogen bond with RBL2 (Fig 4B). In addition, our enzymatic assays indicated that Asp8 is important for the activity of SePSK (Fig 2D). To further verified this result, we measured the binding affinity for D-ribulose of both wild type (WT) and D8A mutant of SePSK using a surface plasmon resonance method [12]. The results showed that the affinity of D8A-SePSK with D-ribulose is weaker than that of WT with a reduction of approx. two third (Fig 4C). Dissociation rate constant (Kd) of wild type and D8A-SePSK are 3 ms^-1^ and 9 ms^-1^, respectively. The results implied that the second RBL binding site plays a role in the D-ribulose kinase function of SePSK. However, considering the high concentration of D-ribulose used for crystal soaking, as well as the relatively weak electron density of RBL2, it is also possible that the second binding site of D-ribulose in SePSK is an artifact.

### Simulated conformational change of SePSK during the catalytic process

It was reported earlier that the crossing angle between the domain I and domain II in FGGY family carbohydrate kinases is different [2, 4, 8, 13, 14]. In addition, this difference may be caused by the binding of substrates and/or ATP. As reported previously, members of the sugar kinase family undergo a conformational change to narrow the crossing angle between two domains and reduce the distance between substrate and ATP in order to facilitate the catalytic reaction of phosphorylation of sugar substrates. After comparing structures of apo-SePSK, RBL-SePSK and AMP-PNP-SePSK, we noticed that these structures presented here are similar. Superposing the structures of RBL-SePSK and AMP-PNP-SePSK, the results show that the nearest distance between AMP-PNP γ-phosphate and RBL1/RBL2 is 7.5 Å (RBL1-O5)/6.7 Å (RBL2-O1) (S8 Fig). This distance is too long to transfer the γ-phosphate group from ATP to the substrate. Since the two domains of SePSK are widely separated in this structure, we hypothesize that our structures of SePSK represent its open form, and that a conformational rearrangement must occur to switch to the closed state in order to facilitate the catalytic process of phosphorylation of sugar substrates.

**Fig 5:**
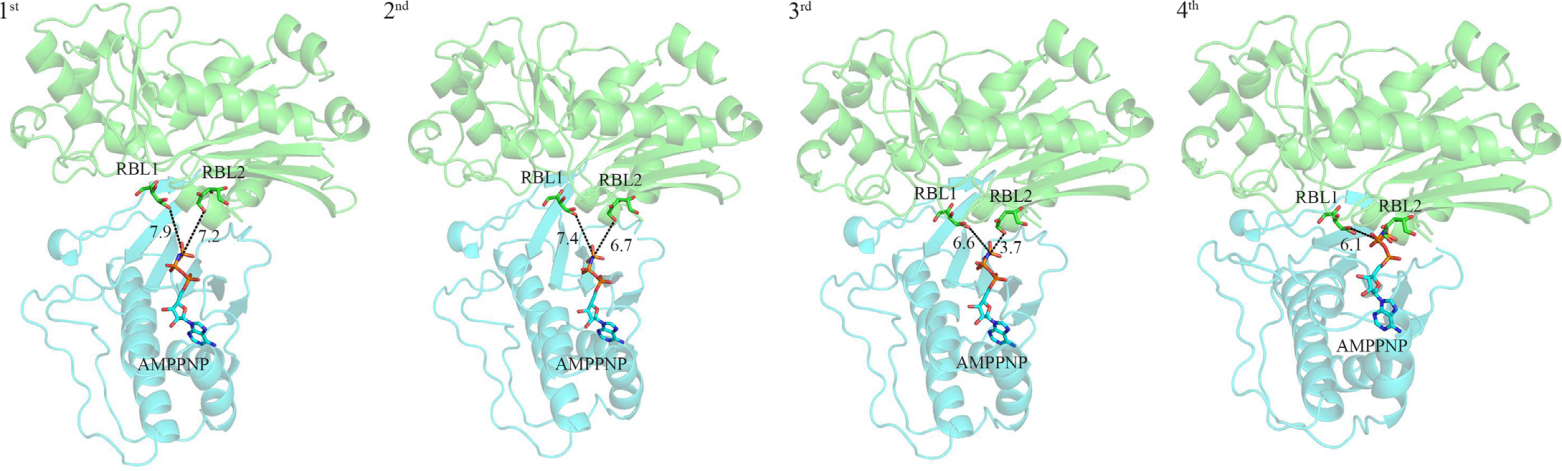
Simulated conformational change of SePSK during the catalytic process. The structures are shown as cartoon and the ligands are shown as sticks. Domain I from D-ribulose-SePSK (green) and Domain II from AMP-PNP-SePSK (cyan) are superposed with apo-AtXK-1 (1^st^), apo-SePSK (2^nd^), 3LL3 (3^rd^) and 1GLJ (4^th^), respectively. The numbers near the black dashed lines show the distances (Å) between two nearest atoms of RBL and AMP-PNP.

For studying such potential conformational change, a simulation on the Hingeprot Server was performed to predict the movement of different SePSK domains [15]. The results showed that domain I and domain II are closer to each other with Ala228 and Thr401 in A2 as Hinge-residues. Based on the above results, SePSK is divided into two rigid parts. The domain I of RBL-SePSK (aa. 1–228, aa. 402–421) and the domain II of AMP-PNP-SePSK (aa. 229–401) were superposed with structures, including apo-AtXK-1, apo-SePSK, xylulose kinase from *Lactobacillus acidophilus* (PDB code: 3LL3) and the S58W mutant form of glycerol kinase from *Escherichia coli* (PDB code: 1GLJ). The results of superposition displayed different crossing angle between these two domains. After superposition, the distances of AMP-PNP γ-phosphate and the fifth hydroxyl group of RBL1 are 7.9 Å (superposed with AtXK-1), 7.4 Å (superposed with SePSK), 6.6 Å (superposed with 3LL3) and 6.1 Å (superposed with 1GLJ). Meanwhile, the distances of AMP-PNP γ-phosphate and the first hydroxyl group of RBL2 are 7.2 Å (superposed with AtXK-1), 6.7 Å (superposed with SePSK), 3.7 Å (superposed with 3LL3), until AMP-PNP γ-phosphate fully contacts RBL2 after superposition with 1GLJ (Fig 5). This distance between RBL2 and AMP-PNP-γ-phosphate is close enough to facilitate phosphate transferring. Together, our superposition results provided snapshots of the conformational changes at different catalytic stages of SePSK and potentially revealed the closed form of SePSK.

In summary, our structural and enzymatic analyses provide evidence that SePSK shows D-ribulose kinase activity, and exhibits the conserved features of FGGY family carbohydrate kinases. Three conserved residues in SePSK were identified to be essential for this function. Our results provide the detailed information about the interaction of SePSK with ATP and substrates. Moreover, structural superposition results enable us to visualize the conformational change of SePSK during the catalytic process. In conclusion, our results provide important information for a more detailed understanding of the mechanisms of SePSK and other members of FGGY family carbohydrate kinases.

## Materials and Methods

### Cloning, expression and purification of SePSK

The gene encoding SePSK was amplified by polymerase chain reaction (PCR) with forward primer 5' CATGCCATGGGCATGGTCGTTGCACTTGGCCTCG 3' containing an internal *Nco* I restriction site (underlined) and reverse primer 5' CCGCTCGAGGGTTCTCTTTAACCCCGCCG 3' including an internal *Xho* I restriction site (underlined) from *Synechococcus elongatus* PCC 7942 genomic DNA. The amplified PCR product was digested with *Nco* I and *Xho* I (Takara) and ligated into linearized pET28-a vector (Novagen) between *Nco* I and *Xho* I restriction sites with a C-terminal his6 tag. The recombinant plasmids were transformed into competent *Escherichia coli Trans*10 cells for DNA production and purification, and the final constructs were verified by sequencing. The recombinant vectors were transformed into *Escherichia coli* BL21 (DE3) to express the protein. After induction with the 1 mM IPTG at 289 K in Luria-Bertani medium until the cell density reached an OD 600 nm of 0.6–0.8, the cells were harvested by centrifugation at 6,000 g at 4°C for 15 min, re-suspended in buffer A (20 mM Tris-HCl, pH 8.0, 500 mM NaCl, 5 mM imidazole) and disrupted by sonication. After centrifuge 40,000 g for 30 min, the protein was purified by passage through a Ni^2+^ affinity column in buffer A, and then washed the unbound protein with buffer B (20 mM Tris-HCl, pH 8.0, 500 mM NaCl, 60 mM imidazole), and eluted the fraction with the buffer C (20 mM Tris-HCl, pH 8.0, 500 mM NaCl, 500 mM imidazole). After that, the protein was further purified by size exclusion chromatography with Superdex 200 10/300 GL (GE Healthcare) equilibrated with the buffer D (20 mM Tris-HCl, pH 8.0, 300 mM NaCl). The eluted major peak fraction was concentrated to 20 mg/mL protein using 10,000 MCWO centrifugal filter units (Millipore) and stored at -80°C for crystallization trials. The purified product was analyzed by SDS-PAGE with a single band visible only.

### Cloning, expression and purification of AtXK-1

The gene encoding AtXK-1 was amplified by PCR using a forward primer 5' TACTTCCAATCCAATGCTGTTATGAGTGGCAATAAAGGAACGA 3' and reverse primer 5' TTATCCACTTCCAATGTTACAAACCACTGTTCTGTTTTGCGCCC 3' from cDNA library of *Arabidopsis thaliana*. The underlined nucleotides were used for the ligation-independent cloning. The PCR product was treated by T4 DNA polymerase (LIC-qualified, Novagen) and then cloned into linearized pMCSG7 vector treated by T4 DNA polymerase (LIC-qualified, Novagen) with an N-terminal his6 tag though ligation-independent cloning method [16]. The final construct was confirmed by DNA sequencing after amplified in competent *Escherichia coli Trans*10 cells. The recombinant vectors were transformed into *Escherichia coli* BL21 (DE3) for protein expression. After induction with 1 mM IPTG at 289 K in Luria-Bertani medium, cells were grown until the cell density reached an OD 600 nm of 0.6–0.8. Subsequent purification was identical to that used for SePSK, except that there was one additional step, during which tobacco etch virus protease was used to digest the crude AtXK-1 protein for removal of the N-terminal his6 tag following Ni^2+^ affinity purification. Ni^2+^ affinity column buffer contained extra 20% glycerol. The protein was further purified by size exclusion chromatography with Superdex 200 10/300 GL (GE Healthcare) in elution buffer consisting of 20 mM HEPES, pH 7.5, 100 mM NaCl. Finally, AtXK-1 protein was concentrated to 40 mg/mL protein using 10,000 MCWO centrifugal filter units (Millipore) and stored at -80°C prior to crystallization trials. Purity was verified by SDS-PAGE with a single band visible only.

### Site-directed mutagenesis of SePSK

The gene of D8A and T11A mutations were amplified by PCR with the forward primer 5' CATGCCATGGGCATGGTCGTTGCACTTGGCCTCGCCTTCGGCAC 3' and forward primer 5' CATGCCATGGGCATGGTCGTTGCACTTGGCCTCGACTTCGGCGCCTCTGGAGCCC 3' (mismatched base pairs are underlined). The reverse primers of D8A and T11A mutants, the further constructions and purification procedures were identical with those used for wild type SePSK.

The N-terminal sequence of D221A was amplified with forward primer (T7 promoter primer) 5' TAATACGACTCACTATA 3' and reverse primer 5' AGCAGCAATGCTAGCCGTTGTACCG 3’, and the C-terminal sequence of D221A was amplified with forward primer 5' TGCCGGTACAACGGCTAGCATTGCT 3' and reverse primer (T7 terminator primer) CGATCAATAACGAGTCGCC (mismatched base pairs are underlined). The second cycle PCR used the above PCR products as templates, and the construction and purification procedures were identical to those used for wild type SePSK.

### Crystallization and data collection

Crystallization trials of SePSK and AtXK-1 were carried out at 281 K by mixing equal volume of 20 mg/ml protein and reservoir solution with the sitting-drop vapor diffusion method. The reservoir solution was PEG Rx I-35 (0.1 M BIS-TRIS pH 6.5, 20% w/v Polyethylene glycol monomethyl ether 5,000) (Hampton research). After 2 or 3 days, the rod-like crystals could be observed. For phasing, the high-quality apo-SePSK crystals were soaked in mother liquor containing 1 mM ethylmercuricthiosalicylic acid, sodium salt (Hampton research, heavy atom kit) overnight at 281 K. In order to get the complexes with ATP and AMP-PNP, the crystals of apo-SePSK and apo-AtXK-1 were incubated with the reservoir including 10 mM ATP and 20 mM MgCl_2_ as well as 10 mM AMP-PNP and 20 mM MgCl_2_, respectively. The apo-SePSK crystals were incubated with the reservoir including 10 mM D-ribulose in order to obtain the complex D-ribulose-bound SePSK (RBL-SePSK). The crystals of three mutants (D8A, T11A and D221A) grew in the same condition as that of the wild type SePSK. The crystals were dipped into reservoir solution supplemented with 15% glycerol and then flash frozen in a nitrogen gas stream at 100 K. All data sets were collected at Shanghai Synchrotron Radiation Facility, Photo Factory in Japan and Institute of Biophysics, Chinese Academy of Sciences. Diffraction data were processed using the HKL2000 package [17].

### Structure determination and refinement

The initial phases of SePSK were obtained from the Hg-derivative crystals by single isomorphous replacement anomalous scattering (SIRAS) using AutoSol from the PHENIX suite [18]. AutoBuild from the PHENIX suite was used to build 75% of the main chain of apo-SePSK, and the remaining residues were built manually by Coot [19]. All other structures were solved by molecular replacement method using apo-SePSK as an initial model. The model was refined using phenix.refine and REFMAC5 [20]. The final model was checked with PROCHECK [21]. All structural figures were prepared by PyMOL [22]. The summary of the data-collection and structure-refinement statistics is shown in Table 1 and S1 Table. Atomic coordinates and structure factors in this article have been deposited in the Protein Data Bank. The deposited codes of all structures listed in the Table 1 and S1 Table.

**Table 1:** Data collection and refinement statistics.

Data set	Hg-SePSK	apo-SePSK	AMP-PNP-SePSK	RBL-SePSK	apo-AtXK-1
Data collection					
Space group	C 1 2 1	C 1 2 1	C 1 2 1	C 1 2 1	P21
Wavelength (Å)	1.54178	1.54178	1.54178	1.54178	1.54178
Cell parameters					
a/b/c(Å)	103.1, 46.6, 88.3	110.2, 49.0, 86.9	103.5, 46.6, 88.0	102.6, 47.0, 88.7	49.7, 87.9, 53.6
α/β/γ(°)	90.0, 91.9, 90.0	90.0, 110.3, 90.0	90.0, 91.0, 90.0	90.0, 91.4, 90.0	90.0, 97.0, 90.0
Resolution (Å)^a^	50.00–2.20(2.28–2.20)	50.00–2.30(2.38–2.30)	50.00–2.30(2.38–2.30)	50.00–2.35(2.43–2.35)	50.00–2.00(2.07–2.00)
R merge^b^	0.105(0.514)	0.149(0.501)	0.082(0.503)	0.095(0.507)	0.106(0.454)
〈 I/σ(I)〉	28.89(4.07)	13.85(4.10)	10.18(1.79)	19.4(4.6)	12.91(4.08)
Completeness (%)	92.3(99.2)	96.1(94.2)	98.9(99.8)	99.8(100.0)	97.1(94.5)
Redundancy	6.7(5.1)	7.4(7.5)	2.4(2.4)	6.9(6.7)	7.2(6.9)
Refinement statistics					
Resolution (Å)		32.501–2.301	24.707–2.300	24.475–2.344	23.771–1.998
R_work_/ R_free_^c^		0.1834/0.2276	0.1975/0.2327	0.2336/0.2687	0.1893/0.2161
No. atoms					
Protein		3503	3196	3209	3256
ligand/ion		-	31	20	-
Water		313	146	143	486
RMSD Bond lengths (Å)^d^		0.003	0.005	0.003	0.003
RMSD Bond angles (°)^d^		0.674	0.886	0.649	0.838
Ramachandran plot (%)					
favoured		98.1	97.8	96.7	99.1
allowed		1.9	2.2	3.3	0.9
disallowed		0.0	0.0	0.0	0.0
PDB code		5HTN	5HTP	5HV7	5HTR

^a^ The values in parentheses correspond to the highest resolution shell.

^b^ R_merge_ = *∑*_*j*_*∑*_*h*_|*I*_*j*,*h*_*-<I*_*h*_*>*|/*∑*_*j*_*∑*_*h*_<*I*_*h*_*>* where *h* are unique reflection indices and I_*j*,*h*_ are intensities of symmetry-related reflections and <*I*_*h*_> is the mean intensity.

^c^ R-work and R-free were calculated as follows: R = Σ (|Fobs-Fcalc|)/Σ |Fobs| ×100, where Fobs and Fcalc are the observed and calculated structure factor amplitudes, respectively.

^d^ Root mean square deviations (r.m.s.d.) from standard values.

### ADP-Glo kinase assay

ADP-Glo kinase assay was used according to the manufacturer’s instructions (Promega) [23]. Each reaction mixture system consisted of 8 uM enzyme, 100 uM ATP, 1 mM MgCl_2_, 20 mM HEPES (pH 7.4), 5 mM substrate. The reaction was initiated by adding the purified enzyme into the reaction system. After incubation at 298 K for different time, equal volume ADP-Glo™ reagent was added to terminate the kinase reaction and to deplete any remaining ATP. Subsequently, kinase detection reagent with double volume of reaction system was added to convert ADP to ATP and allowed the newly synthesized ATP to be measured using a luciferase/luciferin reaction which produced luminescence signal and could be recorded. After incubation at room temperature for another 60 min, luminescence was detected by Varioskan Flash Multimode Reader (Thermo). The reference experiment was carried out in the same reaction system without the enzyme. For each assay, at least three repeats were performed for the calculation of mean values and standard deviations (SDs). The purity of five substrates in the activity assays was ≥98% (D-ribulose, Santa cruz), 99.7% (L-ribulose, Carbosynth), 99.3% (D-xylulose, Carbosynth), 99.5% (L-xylulose, Carbosynth) and 99.0% (Glycerol, AMRESCO).

### Surface plasmon resonance

Surface plasmon resonance (SPR) was used to analyze the interaction of SePSK and D-ribulose. The SPR experiments were performed on a Biacore T100 system using series S CM5 sensor chips (GE Healthcare). All sensorgrams were recorded at 298 K. The proteins in buffer containing 20 mM HEPES, pH 7.5, 100 mM NaCl, was diluted to 40 ug/ml by 10 mM sodium acetate buffer at pH 4.5. All flow cells on a CM5 sensor chip were activated with a freshly prepared solution of 0.2 M 1-ethyl-3-(3-dimethylaminopropyl)-carbodiimide (EDC) and 0.05 M N-hydroxysuccinimide (NHS) in a ratio of 1:1 at a constant flow rate of 10 ul/min for 420 s. Deactivation of the surface was performed with an injection of a 1 M solution of ethanolamine-HCl (pH 8.5) using the same flow rate and duration. Kinetic parameters were derived from data sets acquired in single-cycle mode. Each run consisted of five consecutive analytic injections at 125, 250, 500, 1000 and 2000 uM. Analytic injections lasted for 60 s, separated by 30 s dissociation periods. Each cycle was completed with an extended dissociation period of 300 s. The specific binding to a blank flow cell was subtracted to obtain corrected sensorgrams. Biacore data were analyzed using BiaEvaluation software (GE Healthcare) by fitting to a 1:1 Langmuir binding fitting model [12].

### Accession Codes

Coordinates and structure factors for all the structures in this article have been deposited in the Protein Data Bank. These accession codes are 5HTN, 5HTP, 5HUX, 5HV7, 5HTJ, 5HU2, 5HTY, 5HTR, 5HTV and 5HTX. The corresponding-structures are apo-SePSK, AMP-PNP-SePSK, ADP-SePSK, RBL-SePSK, D8A-SePSK, T11A-SePSK, D221A-SePSK, apo-AtXK1, AMP-PNP-AtXK1 and ADP-AtXK1, respectively.

## Supporting Information

S1 Fig Sequence alignment of SePSK and AtXK-1. Strictly conserved and similar residues are marked with a red background or red letter, respectively. The secondary structure elements are labeled at the top (SePSK) and bottom (AtXK-1).
(PDF)

S2 Fig The similar folding pattern among different FGGY family carbohydrate kinases. Based on the SePSK arrangement, the components of A1, A2, A3, B1 and B2 are assigned. The five superposed FGGY family carbohydrate kinases are shown in different colors, which are putative sugar kinases from *Synechococcus elongatus* PCC 7942: SePSK, xylulose kinase-1 from *Arabidopsis thaliana*: AtXK-1, xylulose kinase from *Escherichia coli*: 2ITM, L-fuculose kinase from *Streptococcus pneumonia*: 4C23 and ribulokinase from *Bacillus halodurans*: 3QDK.
(PDF)

S3 Fig Structural comparison of apo-SePSK and apo-AtXK-1. The similar parts between the two structures are shown in light blue (SePSK) and gray (AtXK-1), while the region with differences are colored by slate (SePSK) and yellow (AtXK-1). In the right panel, the loop3 linking β3 and α4 is bent back to the inner part in SePSK. The distance of the corresponding residues between the two structures is 15.4 Å.
(PDF)

S4 Fig Electron density map of ADP in ADP-SePSK structure. The right panel is a zoom-in view of electron density map of ADP. The structure of ADP-SePSK is shown as violet cartoon and ADP molecule is shown as sticks. The *ǀFoǀ-ǀFcǀ* map of ADP contoured at 3.0 σ is shown in blue mesh.
(PDF)

S5 Fig Structural superposition of ADP-SePSK (pink), AMP-PNP-SePSK (cyan), ADP-AtXK-1 (yellow) and AMP-PNP-AtXK-1 (slate). The ATP binding mode and pocket are similar in all the structures.
(PDF)

S6 Fig The electron density map of RBL1 and RBL2. The RBL-SePSK is shown as green cartoon. The RBL molecules are shown as sticks. The *ǀFoǀ-ǀFcǀ* map contoured at 3.0 σ and the *2ǀFoǀ-ǀFcǀ* map contoured at 0.8 σ are shown in blue and gray mesh.
(PDF)

S7 Fig Comparison of the potential substrate binding pocket between SePSK and 3QDK. Their substrates (RBL1 in SePSK and QDK in 3QDK) are in the same binding cavity. The room mean square deviation (RMSD) of the two structures is 2.30 Å (418 C-alpha of SePSK and 546 C-alpha of 3QDK). RBL-SePSK and 3QDK are colored by green and wheat, respectively. The substrates and coordinated residues are shown as sticks. The hydrogen bond interaction is depicted by the black dashes.
(PDF)

S8 Fig Structural comparison of RBL-SePSK (green) and AMP-PNP-SePSK (cyan). The superposition result shows that the nearest distance between the γ-phosphate group of AMP-PNP and RBL1/RBL2 is 7.5 Å (RBL1-O5)/6.7 Å (RBL2-O1).
(PDF)

S1 Table Data collection and refinement statistics. (PDF)
