# Supplementary material for: Crystal Structures of Putative Sugar Kinases from Synechococcus Elongatus PCC 7942 and Arabidopsis Thaliana
Source: PLoS One. 2016 May 25;11(5):e0156067. doi: 10.1371/journal.pone.0156067 (PMC4880283; doi:10.1371/journal.pone.0156067)
Supplement: S1 Fig — Strictly conserved and similar residues are marked with a red background or red letter, respectively. The secondary structure elements are labeled at the top (SePSK) and bottom (AtXK-1). (PDF) [file pone.0156067.s001.pdf]

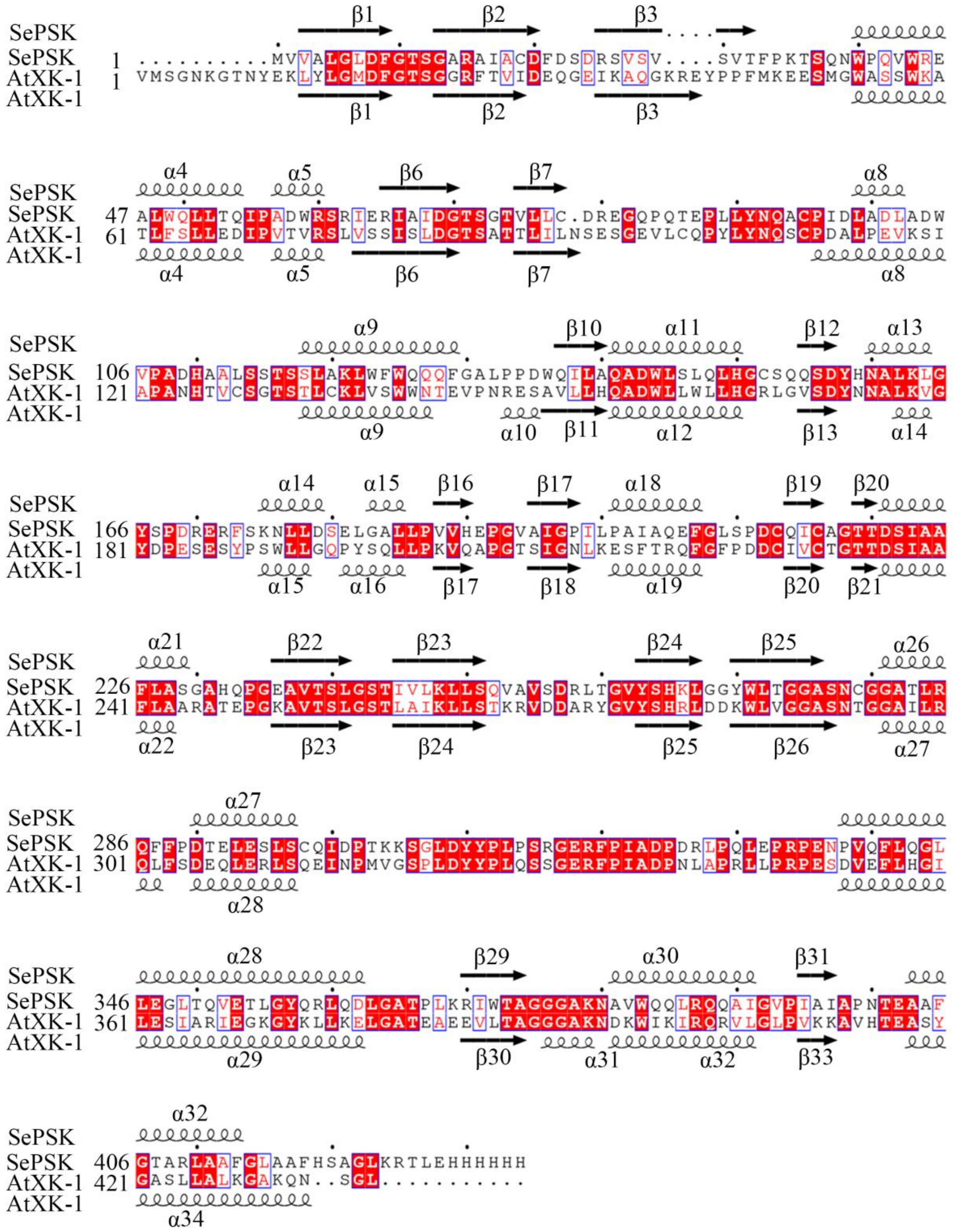

S1 Fig. Sequence alignment of SePSK and AtXK-1. Strictly conserved and similar residues are marked with a red background or red letter, respectively. The secondary structure elements are labeled at the top (SePSK) and bottom (AtXK-1).
