# Supplementary material for: Crystal Structures of Putative Sugar Kinases from Synechococcus Elongatus PCC 7942 and Arabidopsis Thaliana
Source: PLoS One. 2016 May 25;11(5):e0156067. doi: 10.1371/journal.pone.0156067 (PMC4880283; doi:10.1371/journal.pone.0156067)
Supplement: S2 Fig — Based on the SePSK arrangement, the components of A1, A2, A3, B1 and B2 are assigned. The five superposed FGGY family carbohydrate kinases are shown in different colors, which are putative sugar kinases from Synechococcus elongatus PCC 7942: SePSK, xylulose kinase-1 from Arabidopsis thaliana: AtXK-1, xylulose kinase from Escherichia coli: 2ITM, L-fuculose kinase from Streptococcus pneumonia: 4C23 and ribulokinase from Bacillus halodurans: 3QDK. (PDF) [file pone.0156067.s002.pdf]

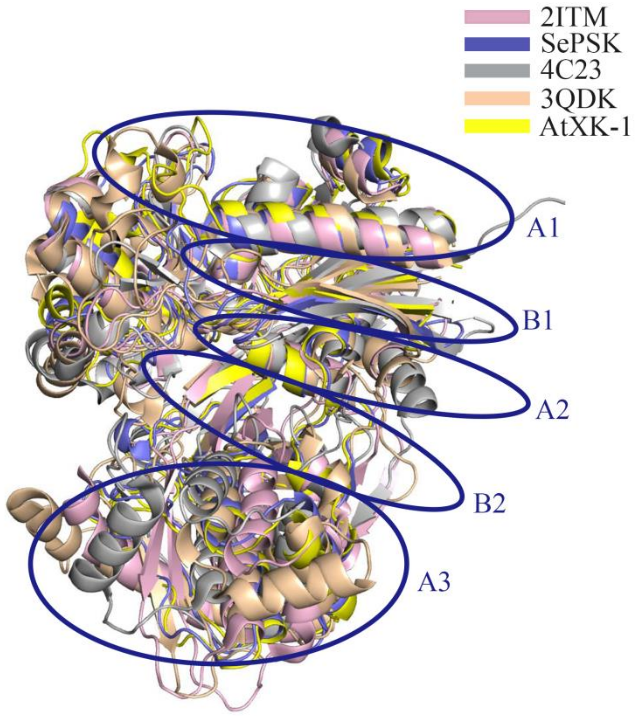

S2 Fig. The similar folding pattern among different FGGY family carbohydrate kinases. Based on the SePSK arrangement, the components of A1, A2, A3, B1 and B2 are assigned. The five superposed FGGY family carbohydrate kinases are shown in different colors, which are putative sugar kinases from *Synechococcus elongatus* PCC 7942: SePSK, xylulose kinase-1 from *Arabidopsis thaliana*: AtXK-1, xylulose kinase from *Escherichia coli*: 2ITM, L-fuculose kinase from *Streptococcus pneumonia*: 4C23 and ribulokinase from *Bacillus halodurans*: 3QDK.
