# Supplementary material for: Crystal Structures of Putative Sugar Kinases from Synechococcus Elongatus PCC 7942 and Arabidopsis Thaliana
Source: PLoS One. 2016 May 25;11(5):e0156067. doi: 10.1371/journal.pone.0156067 (PMC4880283; doi:10.1371/journal.pone.0156067)
Supplement: S3 Fig — The similar parts between the two structures are shown in light blue (SePSK) and gray (AtXK-1), while the region with differences are colored by slate (SePSK) and yellow (AtXK-1). In the right panel, the loop3 linking β3 and α4 is bent back to the inner part in SePSK. The distance of the corresponding residues between the two structures is 15.4 Å. (PDF) [file pone.0156067.s003.pdf]

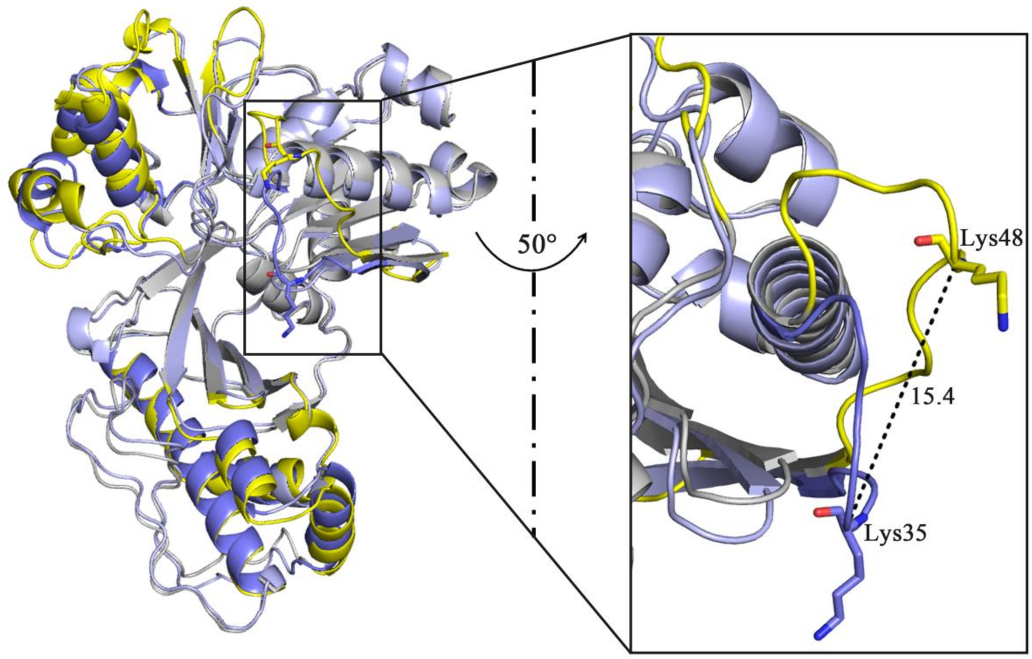

S3 Fig. Structural comparison of apo-SePSK and apo-AtXK-1. The similar parts between the two structures are shown in light blue (SePSK) and gray (AtXK-1), while the region with differences are colored by slate (SePSK) and yellow (AtXK-1). In the right panel, the loop3 linking β3 and α4 is bent back to the inner part in SePSK. The distance of the corresponding residues between the two structures is 15.4 Å.
