# Supplementary material for: Crystal Structures of Putative Sugar Kinases from Synechococcus Elongatus PCC 7942 and Arabidopsis Thaliana
Source: PLoS One. 2016 May 25;11(5):e0156067. doi: 10.1371/journal.pone.0156067 (PMC4880283; doi:10.1371/journal.pone.0156067)
Supplement: S4 Fig — The right panel is a zoom-in view of electron density map of ADP. The structure of ADP-SePSK is shown as violet cartoon and ADP molecule is shown as sticks. The ǀFoǀ-ǀFcǀ map of ADP contoured at 3.0 σ is shown in blue mesh. (PDF) [file pone.0156067.s004.pdf]

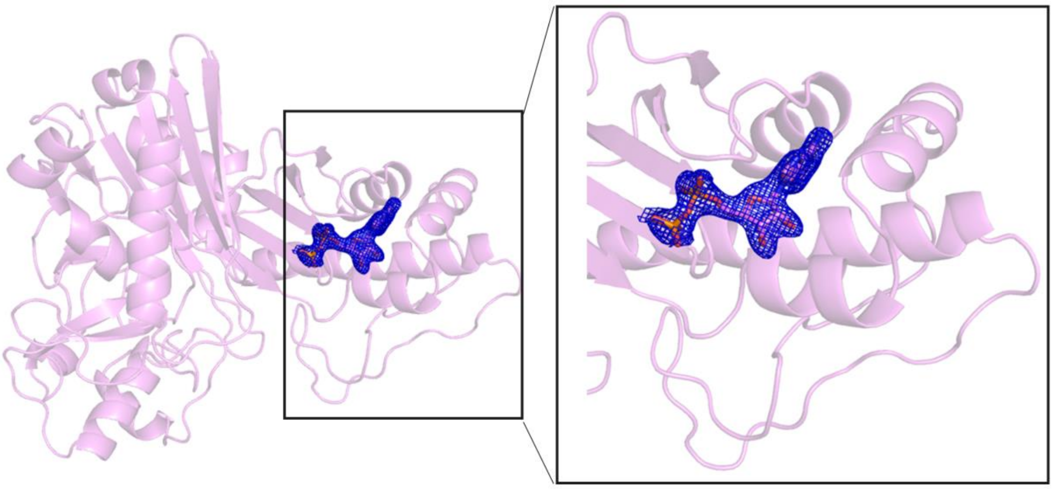

S4 Fig. Electron density map of ADP in ADP-SePSK structure. The right panel is a zoom-in view of electron density map of ADP. The structure of ADP-SePSK is shown as violet cartoon and ADP molecule is shown as sticks. The $|Fo|$-$|Fc|$ map of ADP contoured at 3.0 σ is shown in blue mesh.
