# Supplementary material for: Crystal Structures of Putative Sugar Kinases from Synechococcus Elongatus PCC 7942 and Arabidopsis Thaliana
Source: PLoS One. 2016 May 25;11(5):e0156067. doi: 10.1371/journal.pone.0156067 (PMC4880283; doi:10.1371/journal.pone.0156067)
Supplement: S5 Fig — The ATP binding mode and pocket are similar in all the structures. (PDF) [file pone.0156067.s005.pdf]

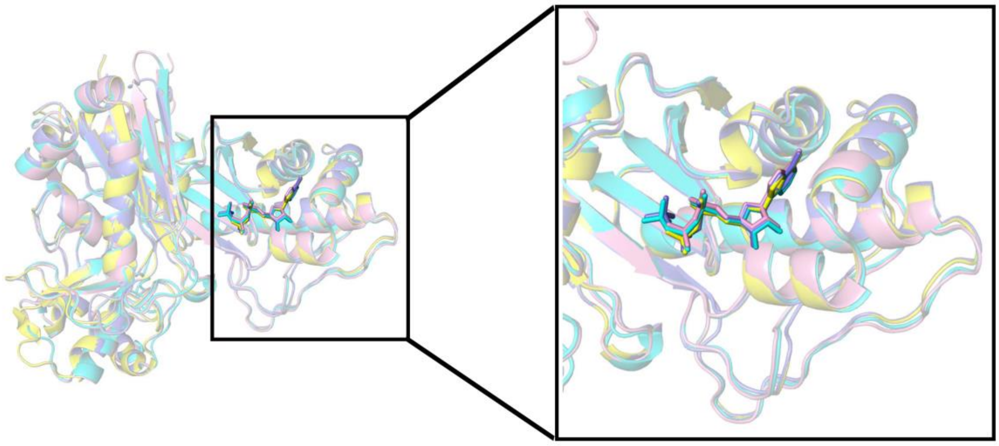

S5 Fig. Structural superposition of ADP-SePSK (pink), AMP-PNP-SePSK (cyan), ADP-AtXK-1 (yellow) and AMP-PNP-AtXK-1 (slate). The ATP binding mode and pocket are similar in all the structures.
