# Supplementary material for: Crystal Structures of Putative Sugar Kinases from Synechococcus Elongatus PCC 7942 and Arabidopsis Thaliana
Source: PLoS One. 2016 May 25;11(5):e0156067. doi: 10.1371/journal.pone.0156067 (PMC4880283; doi:10.1371/journal.pone.0156067)
Supplement: S6 Fig — The RBL-SePSK is shown as green cartoon. The RBL molecules are shown as sticks. The ǀFoǀ-ǀFcǀ map contoured at 3.0 σ and the 2ǀFoǀ-ǀFcǀ map contoured at 0.8 σ are shown in blue and gray mesh. (PDF) [file pone.0156067.s006.pdf]

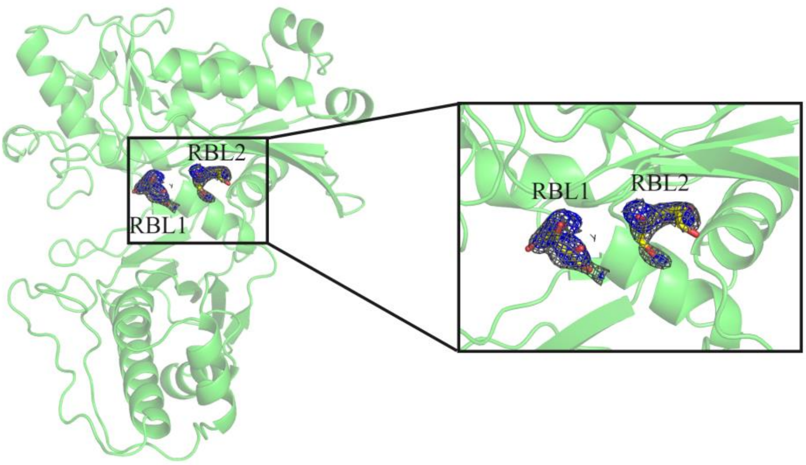

S6 Fig. The electron density map of RBL1 and RBL2. The RBL-SePSK is shown as green cartoon. The RBL molecules are shown as sticks. The |*Fo*|-|*Fc*| map contoured at 3.0 σ and the *2*|*Fo*|-|*Fc*| map contoured at 0.8 σ are shown in blue and gray mesh.
