# Supplementary material for: Crystal Structures of Putative Sugar Kinases from Synechococcus Elongatus PCC 7942 and Arabidopsis Thaliana
Source: PLoS One. 2016 May 25;11(5):e0156067. doi: 10.1371/journal.pone.0156067 (PMC4880283; doi:10.1371/journal.pone.0156067)
Supplement: S7 Fig — Their substrates (RBL1 in SePSK and QDK in 3QDK) are in the same binding cavity. The room mean square deviation (RMSD) of the two structures is 2.30 Å (418 C-alpha of SePSK and 546 C-alpha of 3QDK). RBL-SePSK and 3QDK are colored by green and wheat, respectively. The substrates and coordinated residues are shown as sticks. The hydrogen bond interaction is depicted by the black dashes. (PDF) [file pone.0156067.s007.pdf]

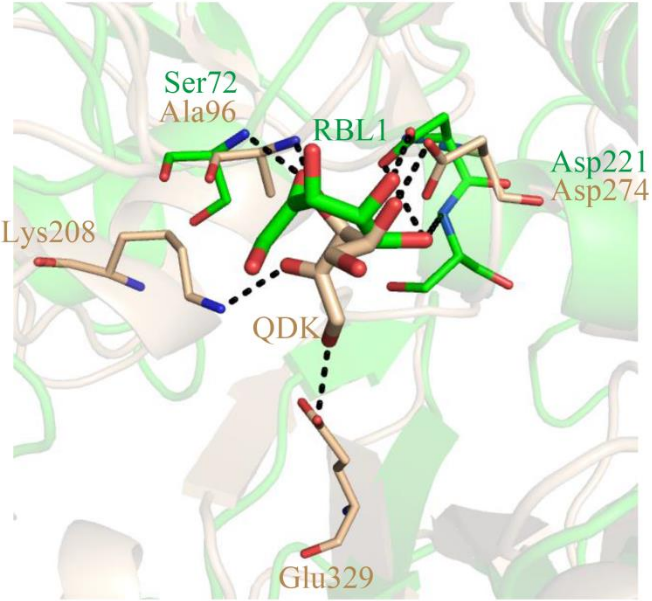

S7 Fig. Comparison of the potential substrate binding pocket between SePSK and 3QDK. Their substrates (RBL1 in SePSK and QDK in 3QDK) are in the same binding cavity. The room mean square deviation (RMSD) of the two structures is 2.30 Å (418 C-alpha of SePSK and 546 C-alpha of 3QDK). RBL-SePSK and 3QDK are colored by green and wheat, respectively. The substrates and coordinated residues are shown as sticks. The hydrogen bond interaction is depicted by the black dashes.
