# Supplementary material for: Crystal Structures of Putative Sugar Kinases from Synechococcus Elongatus PCC 7942 and Arabidopsis Thaliana
Source: PLoS One. 2016 May 25;11(5):e0156067. doi: 10.1371/journal.pone.0156067 (PMC4880283; doi:10.1371/journal.pone.0156067)
Supplement: S8 Fig — The superposition result shows that the nearest distance between the γ-phosphate group of AMP-PNP and RBL1/RBL2 is 7.5 Å (RBL1-O5)/6.7 Å (RBL2-O1). (PDF) [file pone.0156067.s008.pdf]

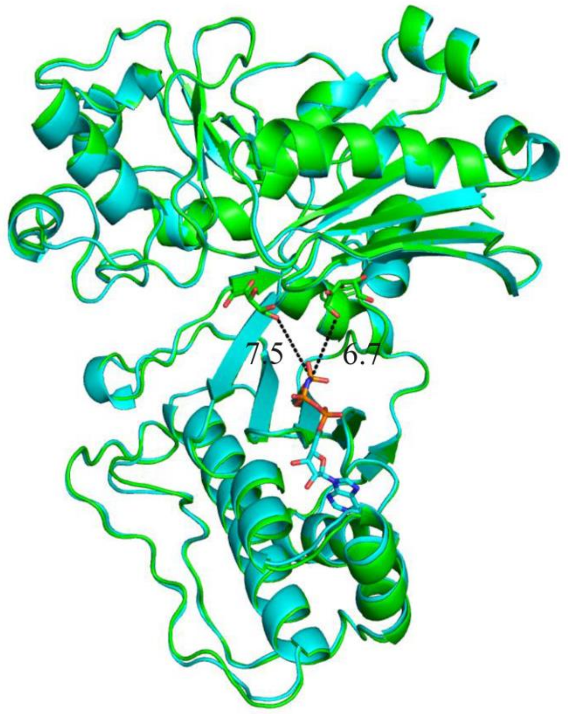

S8 Fig. Structural comparison of RBL-SePSK (green) and AMP-PNP-SePSK (cyan). The superposition result shows that the nearest distance between the γ-phosphate group of AMP-PNP and RBL1/RBL2 is 7.5 Å (RBL1-O5)/6.7 Å (RBL2-O1).
