# Supplementary material for: Crystal Structures of Putative Sugar Kinases from Synechococcus Elongatus PCC 7942 and Arabidopsis Thaliana
Source: PLoS One. 2016 May 25;11(5):e0156067. doi: 10.1371/journal.pone.0156067 (PMC4880283; doi:10.1371/journal.pone.0156067)
Supplement: S1 Table — (PDF) [file pone.0156067.s009.pdf]

S1 Table. Data collection and refinement statistics

<table>
<tr><td colspan="2">Data set</td><td>ADP-SePSK</td><td>T11A-SePSK</td><td>D221A-SePSK</td><td>D8A-SePSK</td><td>AMP-PNP-AtXK-1</td><td>ADP-AtXK-1</td></tr>
<tr><td colspan="2">Data collection</td><td></td><td></td><td></td><td></td><td></td><td></td></tr>
<tr><td colspan="2">Space group</td><td>C 1 2 1</td><td>C 1 2 1</td><td>C 1 2 1</td><td>C 1 2 1</td><td>P21</td><td>P21</td></tr>
<tr><td colspan="2">Wavelength (Å)</td><td>1.00</td><td>1.54178</td><td>0.97916</td><td>0.97916</td><td>0.97917</td><td>0.97917</td></tr>
<tr><td rowspan="6">Cell parameters</td><td>a (Å)</td><td>101.104</td><td>98.926</td><td>97.887</td><td>101.942</td><td>49.565</td><td>49.529</td></tr>
<tr><td>b (Å)</td><td>46.662</td><td>46.686</td><td>47.206</td><td>46.920</td><td>87.454</td><td>87.489</td></tr>
<tr><td>c (Å)</td><td>89.394</td><td>89.617</td><td>89.835</td><td>88.644</td><td>53.364</td><td>53.360</td></tr>
<tr><td>α(°)</td><td>90.000</td><td>90.000</td><td>90.000</td><td>90.000</td><td>90.000</td><td>90.000</td></tr>
<tr><td>β(°)</td><td>90.560</td><td>91.385</td><td>91.585</td><td>91.353</td><td>96.780</td><td>97.284</td></tr>
<tr><td>γ(°)</td><td>90.000</td><td>90.000</td><td>90.000</td><td>90.000</td><td>90.000</td><td>90.000</td></tr>
<tr><td colspan="2">Resolution (Å)</td><td>50.00-1.96
(2.03-1.96)</td><td>50.00-2.59
(2.68-2.59)</td><td>50.00-2.80
(2.90-2.80)</td><td>50.00-1.97
(2.04-1.97)</td><td>50.00-1.75
(1.84-1.75)</td><td>50.00-1.49
(1.54-1.49)</td></tr>
<tr><td colspan="2">Rmerge#</td><td>0.075(0.233)</td><td>0.128(0.479)</td><td>0.115(0.427)</td><td>0.056(0.410)</td><td>0.073(0.600)</td><td>0.044(0.301)</td></tr>
<tr><td colspan="2">〈 I/σ(I)〉</td><td>18.80(5.75)</td><td>15.35(4.26)</td><td>12.93(5.48)</td><td>20.79(4.63)</td><td>17.94(3.91)</td><td>27.37(5.26)</td></tr>
<tr><td colspan="2">Completeness (%)</td><td>99.0(98.0)</td><td>99.9(100.0)</td><td>99.9(100.0)</td><td>99.9(100.0)</td><td>99.9(100.0)</td><td>96.3(90.0)</td></tr>
<tr><td colspan="2">Redundancy</td><td>5.1(4.1)</td><td>7.2(7.0)</td><td>7.0(7.4)</td><td>7.3(7.2)</td><td>4.1(4.2)</td><td>4.2(4.1)</td></tr>
<tr><td colspan="2">Refinement statistics</td><td></td><td></td><td></td><td></td><td></td><td></td></tr>
<tr><td colspan="2">Resolution (Å)</td><td>38.345-1.958</td><td>33.605-2.599</td><td>32.634-2.815</td><td>33.834-2.001</td><td>38.395-1.752</td><td>42.835-1.492</td></tr>
<tr><td colspan="2">No. reflections</td><td>29849</td><td>12792</td><td>10011</td><td>28509</td><td>45067</td><td>70724</td></tr>
<tr><td colspan="2">Rwork/ Rfree</td><td>0.1772/0.2246</td><td>0.2065/0.2463</td><td>0.2242/0.2587</td><td>0.1929/0.2232</td><td>0.1712/0.2041</td><td>0.1448/0.1720</td></tr>
<tr><td rowspan="3">No. atoms</td><td>Protein</td><td>3275</td><td>3199</td><td>3198</td><td>3193</td><td>3256</td><td>3256</td></tr>
<tr><td>ligand/ion</td><td>27</td><td>-</td><td>-</td><td>-</td><td>31</td><td>39</td></tr>
<tr><td>Water</td><td>293</td><td>111</td><td>-</td><td>356</td><td>324</td><td>292</td></tr>
<tr><td rowspan="3">B-factors</td><td>Protein</td><td>28.461</td><td>34.319</td><td>36.504</td><td>27.305</td><td>16.145</td><td>16.155</td></tr>
<tr><td>ligand/ion</td><td>33.150</td><td>-</td><td>-</td><td>-</td><td>37.744</td><td>26.297</td></tr>
<tr><td>Water</td><td>34.099</td><td>33.236</td><td>-</td><td>37.072</td><td>26.604</td><td>25.822</td></tr>
<tr><td colspan="2">RMSD Bond lengths (Å)</td><td>0.005</td><td>0.002</td><td>0.002</td><td>0.003</td><td>0.007</td><td>0.014</td></tr>
<tr><td colspan="2">RMSD Bond angles (°)</td><td>0.956</td><td>0.646</td><td>0.616</td><td>0.704</td><td>1.145</td><td>1.453</td></tr>
<tr><td rowspan="3">Ramachandran plot (%)</td><td>favoured</td><td>98.1</td><td>97.6</td><td>98.1</td><td>97.6</td><td>99.1</td><td>99.1</td></tr>
<tr><td>allowed</td><td>1.9</td><td>2.4</td><td>1.9</td><td>2.4</td><td>0.9</td><td>0.9</td></tr>
<tr><td>disallowed</td><td>0.0</td><td>0.0</td><td>0.0</td><td>0.0</td><td>0.0</td><td>0.0</td></tr>
<tr><td colspan="2">PDB code</td><td>5HUX</td><td>5HU2</td><td>5HTY</td><td>5HTJ</td><td>5HTV</td><td>5HTX</td></tr>
</table>
